# Taking the Perfect Selfie: Investigating the Impact of Perspective on the Perception of Higher Cognitive Variables

**DOI:** 10.3389/fpsyg.2017.00971

**Published:** 2017-06-09

**Authors:** Tobias M. Schneider, Claus-Christian Carbon

**Affiliations:** ^1^Department of General Psychology and Methodology, University of BambergBamberg, Germany; ^2^Bamberg Graduate School of Affective and Cognitive Sciences, University of BambergBamberg, Germany; ^3^Research Group EPÆG (Ergonomics, Psychological Æsthetics, Gestalt)Bamberg, Germany

**Keywords:** selfie, viewing perspective, personality assessment, optimization, height-weight illusion, perspective, perception bias, face processing

## Abstract

Taking selfies is now becoming a standard human habit. However, as a social phenomenon, research is still in the fledgling stage and the scientific framework is sparse. Selfies allow us to share social information with others in a compact format. Furthermore, we are able to control important photographic and compositional aspects, such as perspective, which have a strong impact on the assessment of a face (e.g., demonstrated by the height-weight illusion, effects of gaze direction, faceism-index). In Study 1, we focused on the impact of perspective (left/right hemiface, above/below vs. frontal presentation) on higher cognitive variables and let 172 participants rate the perceived attractiveness, helpfulness, sympathy, dominance, distinctiveness, and intelligence, plus important information on health issues (e.g., body weight), on the basis of 14 3D faces. We could show that lateral snapshots yielded higher ratings for attractiveness compared to the classical frontal view. However, this effect was more pronounced for left hemifaces and especially female faces. Compared to the frontal condition, 30° right hemifaces were rated as more helpful, but only for female faces while faces viewed from above were perceived as significant *less* helpful. Direct comparison between *left* vs. *right* hemifaces revealed no effect. Relating to sympathy, we only found a significant effect for 30° right *male* hemifaces, but only in comparison to the frontal condition. Furthermore, female 30° *right hemifaces* were perceived as more intelligent. Relating to body weight, we replicated the so-called “height-weight illusion.” Other variables remained unaffected. In Study 2, we investigated the impact of a typical selfie-style condition by presenting the respective faces from a lateral (left/right) and tilted (lower/higher) vantage point. Most importantly, depending on what persons wish to express with a selfie, a systematic change of perspective can strongly optimize their message; e.g., increasing their attractiveness by shooting from above left, and in contrast, decreasing their expressed helpfulness by shooting from below. We could further extent past findings relating to the height-weight illusion and showed that an *additional* rotation of the camera positively affected the perception of body weight (lower body weight). We discuss potential explanations for perspective-related effects, especially gender-related ones.

## Introduction

Taking selfies is a well-known but still poorly investigated social phenomenon. In contrast to a classical portrait, it refers to a self-portrait picture taken by ourselves using e.g., the frontal camera of a smartphone and allows us to control important photographic and compositional aspects such as perspective, which has a strong impact on perceptual factors (e.g., variation of the assessed weight, the so-called “height-weight illusion”, see Schneider et al., 2012). It is assumed that taking selfies has now become an important social phenomenon for expressing individual values and personality traits, showing off and sharing the current mood (see e.g., Sorokowska et al., 2016). Despite the high degree of relevance, there is only sparse research that has investigated whether selfies and related self-portraits serve as a valid predictor for personal traits (see e.g., Qiu et al., 2015; Teijeiro-Mosquera et al., 2015). More precisely, the “*nature of selfies*” is not well-investigated: It is suggested that viewing perspective/head rotation and picture details make a selfie different to a classical portrait (see e.g., Bruno and Bertamini, 2013; Bruno et al., 2014; Yeh and Lin, 2014). Other research relating to selfies has revealed that they can serve as valid cues for a respective person's personality traits (Qiu et al., 2015). More precisely, Guntuku et al. (2015) analyzed several visual cues (so-called “mid-level cues”) relating to the selfie-taker's personality (such as facial expression, photo location, Photoshop editing, amount of body visible etc.) and found that *Agreeableness—*in the sense of the *Big-Five* personality factors which are described as personality traits manifesting themselves in individual behavioral characteristics that are perceived as *kind, sympathetic, cooperative, warm*, and *considerate—*(see Thompson, 2008) was negatively correlated with camera height (agreeable individuals are more likely to take selfies from below). They further found that *Conscientiousness—*in the sense of the *Big-Five* which are described as personality traits manifesting themselves in individual behavioral characteristics such as being *neat* and *systematic*; also including such elements as *carefulness, thoroughness, and deliberation—*(see Thompson, 2008) was negatively correlated with private locations. Guntuku et al. (2015) argue that conscientious people do not like to expose their private space in the background. The authors further revealed that Neuroticism is negatively correlated with a duckface expression. However, a clear conclusion based on this resulting data pattern remains unclear. Evidence from research investigating whether faces provide valid predictions about personality related variables suggests that people seem to have high interrater consensus (in case of frontal facial presentations), but only when context information (e.g., expression, clothing, background, or speech) is visible. For example, Nestler et al. (2012) used standardized photographs and demonstrated that *extraversion—*in the sense of the *Big-Five* personality factors which are described as personality traits manifesting themselves in individual behavioral characteristics that are perceived as *outgoing, talkative*, and *energetic behaviors—*(see Thompson, 2008) is associated with facial *attractiveness*, while *openness—*what could be described by six dimensions or facets (of the *Big-Five* personality factors) including *active imagination (fantasy), aesthetic sensitivity, attentiveness to inner feelings, preference for variety*, and *intellectual curiosity* (see Costa and McCrae, 1992)—is associated with the volume of the lips, and *conscientiousness* is associated with facial *femininity*. In another study using full-body images Naumann et al. (2009) demonstrated that also for spontaneous poses and facial expression (in contrast to standardized photographs), observers made quite accurate predictions of the target's personality. Furthermore, also dynamic cues, such as clothing, provided valuable information for the predicted personality (see e.g., Penton-Voak et al., 2001; Qiu et al., 2015 for futher investigations).

For optimizing selfies in terms of what the depictions show in regard of higher cognitive variables, we might use specific perspectives—a method established for a very long time in the field of classical portrait photography. It was shown within a wide spectrum of research approaches that such higher cognitive variables can change the attitude and behavior toward the depicted person. For instance, facial *attractiveness* was revealed to positively affect gaze behavior (e.g., longer gaze duration, larger cone of gaze etc.) in human beings (see e.g., Maner et al., 2003, 2007; Leder et al., 2010, 2016; van Straaten et al., 2010; Baranowski et al., 2016). Research in the field of social psychology revealed that attractive individuals are perceived as more socially capable, popular, and competent (Dion et al., 1972; Eagly et al., 1991). They further earn more wages (Mobius and Rosenblat, 2006; Toledano, 2012), are even more likely to win political elections (Banducci et al., 2008; King and Leigh, 2009; Berggren et al., 2010), are sentenced more lenient by courts (Stewart, 1980), and are associated with a higher level of bodily health (Jones et al., 2001; Rhodes et al., 2001; Fink et al., 2006; Nedelec and Beaver, 2014).

However, past research identified perceived facial *attractiveness, masculinity*, and *dominance* as important cues to sexual fitness bodily health in male individuals, even if the manner how they interact remains inconsistent (see Penton-Voak et al., 2001 for a review). Masculine facial features (e.g., large jaws and prominent brows) in males are suggested to be testosterone dependent and therefore associated with greater immunocompetence, phenotypic and genetic quality, respectively (see e.g., Folstad and Karter, 1992; Thornhill and Grammer, 1999). On the one hand, Cunningham et al. (1990) as well as Grammer and Thornhill (1994) demonstrated that masculine facial features are preferred by female observers, while facial masculinity is highly related to the perceived *dominance* in male faces across female and male observers (see e.g., McArthur and Apatow, 1984; McArthur and Berry, 1987; Berry and Brownlow, 1989; Perrett et al., 1998). On the other hand, perceived *dominance* is highly correlated with associated muscle mass (Frederick and Haselton, 2007), as well as a higher level of testosterone (Swaddle and Reierson, 2002) in male individuals. However, scientific reports about direct effects of *dominance* on the perceived *attractiveness* are rather inconsistent, for example, positive effects are reported by e.g., Keating (1985), but see Perrett et al. (1998) for reported negative effects. With respect to viewing perspective and the perception of the associated *dominance* on the basis of faces, there is evidence that raising the head improves the perception of perceived dominance (e.g., Otta et al., 1994; Mignault and Chaudhuri, 2003; Chiao et al., 2008; Rule et al., 2012). Furthermore, Burke and Sulikowski (2010) revealed a strong relationship between upward postures and perceived *masculinity*. Results from studies investigating effects of facial lateralization (*left hemiface* which is from the owner's perspective the left side of the face vs. *right hemiface* which is from the owner's perspective the right side of the face) with chimaeric faces (combining one side of a face and mirroring it to the other side) revealed that the *right hemiface* is associated with higher ratings of *attractiveness* (see e.g., Zaidel et al., 1995; Burt and Perrett, 1997; but see Zaidel and Cohen, 2005 who only found effects for female faces). Following these results, we strongly expect that the showing the right cheek (*right hemiface*) positively affects the perceived *attractiveness* of a face. Furthermore, a face that is viewed from a *lower* vantage point should be perceived as more *dominant*.

Relating to female individuals, Jones (1995) revealed that faces that appear to be younger than the actual age (neotenous faces e.g., small lower jaw and nose, and large lips) are rated as more attractive by male raters across five populations. In a further experiment, Jones demonstrated that manipulation of facial features toward increased neoteny resulted in higher ratings of attractiveness. From an evolutionary perspective, preferring female youthful facial features by male individual was more adaptive since neoteny is highly associated with greater fertility, fecundity, phenotypic and genetic quality (see e.g., Thornhill and Gangestad, 1993; Perrett et al., 1998). Beside the fact that (primarily for female faces) the *right hemiface* is associated with higher perceived attractiveness, there is also evidence for lateralization effects on the perceived age. For example, Burt and Perrett (1997) revealed a *right hemiface* bias, hence the perceived age of the face is biased toward the *right hemiface*. Similarly, Hole and George (2011) suggested that holistic face processing (in the sense that facial parts are bound into a single “Gestalt,” see Tanaka and Farah, 1993) plays an important role in age perception. Using the so-called “composite face effect” (assembling the top half of one face with the bottom half of a different face produces the impression of a “new” face) they asked participants to estimate the associated age of a composite face and found that participants' estimates were significantly biased toward the age of the bottom half of the face. Regarding direct changes of viewing perspective (or head posture), downward pitched heads appear to be younger and upward pitched heads appear to be older (Bruce et al., 1989). According to past research, we hypothesize a positive effect for the *right hemiface* on the perceived *attractiveness* also for female faces. Furthermore, with respect to the aforementioned relationship between perceived younger age and higher ratings of attractiveness in female faces, we cautiously assume that a downward pitched female face is associated with higher ratings of attractiveness.

There is also research on more “objective” variables relating to the actual *health* such as the body height and weight of the “selfied” person. It is scientifically recognized that body shape and mass is highly related to the associated health (e.g., Swami and Tovee, 2005, 2008; Furnham et al., 2006; Tovee et al., 2006) and faces also provide valid cues to body weight and health. For example, Coetzee et al. (2009), Coetzee et al. (2010), as well as Tinlin et al. (2013) demonstrated that facial adiposity could be taken as a predictor of various health related variables, such as the associated immunological competence, cardiovascular function, frequency of respiratory infections, and ultimate mortality. Furthermore, facial adiposity is also highly correlated with the perceived attractiveness (Re and Rule, 2016). Viewing perspective, which is often used as a composition property in selfies, strongly affects the perception of these variables (Schneider et al., 2012, 2013). However, with respect to lateralization effects on the perceived health on the basis of faces, there is scientific disagreement (right side of the face see e.g., Reis and Zaidel, 2001; Kramer and Ward, 2011; Jones et al., 2012; but see Sitton et al., 2006 for the left side of the face).

In fact, many people use perspective as a powerful technique to enhance or optimize some further (non- health- and mating-related) properties. Whether this is done implicitly or explicitly, it is clear that perspective is very differently employed in selfies and classical portraits (Bruno and Bertamini, 2013; Bruno et al., 2014). It is assumed that turning the face to the right (showing the left cheek: *left hemiface*) affects the perception of some emotions. More precisely, the left side of the face was rated as more emotionally expressive and emotions were perceived more intense (see e.g., Sackeim et al., 1978; Zaidel et al., 1995; Nicholls et al., 2002; Jones et al., 2012; Lindell, 2013a,b; Low and Lindell, 2016). This is widely in accordance with findings that the left cheek is overrepresented in classical portraits, see e.g., Bruno and Bertamini (2013) and McManus and Humphrey (1973); but also see contrasting research by Lindell (2016) who worked on specific cases of art history (i.e., Vincent Van Gogh's work). However, there are still some contradictions about the lateralization of perceptual aspects (e.g., the perception of higher-cognitive variables), for example, see Burt and Perrett (1997) or Jones et al. (2012). More precisely, there is some evidence for the asymmetrical facial organization of these variables. For example, as aforementioned, the right side of the face (*right hemiface*) affects the perception of attractiveness, sex and age, participants gaze at the right side of the face longer, whereas, the left side is perceived as more emotional and more expressive (see e.g., Sackeim et al., 1978; Burt and Perrett, 1997; Nicholls et al., 2002; Butler et al., 2005; Lindell, 2013a,b). Due to the importance of lateral effects and the non-consistent findings reported in the literature, we have made an overview of lateralization effects on a variety of face-relevant variables in Table 1. That there are contradictory findings between face research and empirical findings from the domain of selfies might underline the hypothesis by Bruno et al. (2014) that selfies show a general and systematic deviation from known principles of photographic compositions.

**Table 1:** List of research which investigated the effect of *hemiface* (left vs. right) on the perception of *attractiveness, emotional expression* (posed and spontaneous), *personality related variables*, and *health*, showing that the results are quite far from consistent (emotional expression shows highly consistent results).

**Investigated variable**	**Study**	***N***	**Lateralization effect^#^**
Attractiveness	Burt and Perrett, 1997	132 (73 female)	**Right**
	Dunstan and Lindell, 2012	192 (129 female)	**Right** (♀^*^/♂^n.s.^)
	Sitton et al., 2006	40	**Left**
	Zaidel and Cohen, 2005	27 (15 female)/21 (14 female)	No effect for attractive faces
	Zaidel et al., 1995	26 (16 female)	**Right** (♀^*^/♂^n.s.^)
Emotional expression—posed	Borod et al., 1988	16 (0 female)	**Left**: happiness, surprise, sexual arousal, disgust, fear, anger, confusion, neutral
	Ekman et al., 1981	36	**Left**: smiling
	Indersmitten and Gur, 2003	38 (19 female)	**Left**: happiness, sadness, fear
	Kowner, 1995	72 (36 female)	**Left**: smiling
	Low and Lindell, 2016	90 (70 female)	**Left**: happiness
	Moreno et al., 1990	90	**Left**: smiling
	Nicholls et al., 2002	348 (274 female)	**Left**: general more emotional expressive
	Sackeim et al., 1978	86 (29 female)	**Left**: neutral, sad, anger, fear, surprise, disgust, happy
	Zaidel et al., 1995	18 (9 female)	**Left** (♀^*^/♂^*^): smiling
Emotional expression—spontaneous	Cacioppo and Petty, 1981	50	**Left**: sadness
	Dopson et al., 1984	34 (31 female)	**Left**: happy, sad
	Indersmitten and Gur, 2003	38 (19 female)	**Right**: anger
Personality-related variables	Jones et al., 2012	44 (25 female)	**Right**: general higher accuracy
	Kramer and Ward, 2011	32 (25 female)	**Right**: general higher accuracy
	Okubo et al., 2013	100 (50 female)	**Left**: trustworthiness (smiling faces)
Health	Reis and Zaidel (2001)	24 (12 female)	**Left**
	Sitton et al. (2006)	40	**Right**

# *Left, significant higher ratings for the left side of the face from owner's perspective (left hemiface)*.

*Right, significant higher ratings for the right side of the face from the owner's perspective (right hemiface)*.

* *Controlled for gender, effect was significant*.

The aim of the present study was to provide fundamental information what impact a change of *perspective* has on a variety of higher-order variables that are relevant for expressing personality and for mating. To the authors' knowledge, there is no systematic investigation of how *viewing perspective* affects the perception of higher cognitive variables (such as personality variables) on basis of faces, especially for more selfie-style conditions. Accordingly, we decided to use systematically varied full 3D models which have a clear advantage over typical analysis of selfie-photographs. The factor of is not confounded with other variables such as emotional expression, style, context etc. and therefore, this fundamental information can be easily transferred to statements about selfies. We investigated the impact of systematically manipulated *viewing perspectives* (see method section) on seven social- as well as health- and mating-relevant (so called higher cognitive) variables. First of all, we investigated *attractiveness, dominance, intelligence*, and *body weight* as important predictors to bodily health and fitness.

Secondly, past research in the field of social psychology has identified *helpfulness* or helping behavior as an important social variable. Helping behavior (or helpfulness) as a subcategory of prosocial behavior is intentional and it benefits another living being or group (Hogg and Vaughan, 2013). According to the question of the philosopher Turner (2005), whether altruistic and helpful behavior is an anomaly in human beings, there is a great debate across social psychologists (e.g., Campbell, 1975), sociobiologists (e.g., Wilson, 2000), and evolutionary social psychologists (e.g., Neuberg et al., 2010). The core question seems to be: is altruistic and helpful behavior a trait that has evolutionary survival value? From a raw biological view, altruistic and helpful behavior is associated with non-profitable enhancement of the reproductive fitness of another organism at one's own charge. Turner (2005, p. 317) further asked: “…how could natural selection ever smile upon organisms that sacrifice their own reproductive fitness for another's benefit?” However, this behavior is also empirically observable in animals which underlines the evolutionary importance of it: for example, some types of fishes enter the mouths of their hosts to remove parasites even at mortal danger (Stevens et al., 2005). From a more social psychological view, the apparent benefit of helpful behavior in social groups is well-documented in research (for example, the *bystander intervention*, whereby a person breaks out of the role of a bystander and helps another person in an emergency). Another finding is provided by Baumeister et al. (1988) who revealed a relationship between leadership and helping behavior. Leaders seem to have a generalized responsibility providing a buffer against the diffusion of responsibility.

Thirdly, *sympathy* as another important construct in social psychology. Empathy and *sympathy* are often used interchangeably. However, these terms have distinct meanings (Lishner et al., 2011). One definition of empathy is provided by Hogg and Vaughan (2013) who suggest that it is the ability to experiencing another person's emotions, thoughts and mindset. In contrast, *sympathy* is defined as a feeling of caring about someone else's trouble, sorrow or misfortune, but not necessarily the feeling of sharing the same feelings of another person. It could further be understood as a state of sharing the same interests, attitudes, goals etc. with another person. With respect to mating-related behavior (such as mating choice), research revealed *sympathy* as an important variable. In accordance with the so-called “homogamy hypothesis”, people tend to seek for partners with similar hobbies, habits, interests, attitudes (e.g., religiosity) and mindsets (e.g., Hahn and Blass, 1997; Watson et al., 2004; Luo and Klohnen, 2005; Perry, 2015).

*Distinctiveness*. Carbon et al. (2010) pointed out that this term is somewhat ambiguously defined in research. Following the definition of Wickham and Morris (2003), *distinctiveness* can “traditionally” mean “standing out from a crowd” or, alternatively, “deviating from the average face” (so-called “deviation”). In the present paper, we used the traditional definition from face research with *distinctiveness* as an assessment of the salience of a face standing out of a crowd (of other faces). With respect to research in the field of perceived attractiveness and mating behavior, there is some evidence that *symmetry*, but also *averageness* could be taken as a predictor to bodily health (see e.g., Thornhill and Gangestad, 1993; Grammer and Thornhill, 1994; Shackelford and Larsen, 1997; Jones et al., 2001; Penton-Voak et al., 2001; Rhodes et al., 2001; Zaidel and Cohen, 2005; Fink et al., 2006). According to Valentine's (1991) so-called *Multidimensional Face Space Model*, typical faces (e.g., high level of averageness) are densely located near the centroid of this face space, hence these faces are highly similar; whereas distinctive faces are less densely clustered (Valentine, 1991; Newell et al., 1999). Thus, potential effects of rarely changes in viewing perspective on the perceived *distinctiveness* could be applied to selfie-related techniques.

The finding of evidence that viewing perspective has a great impact could lead to a better understanding of *how* a selfie should be taken and *how* we perceive a given face.

## Study 1

### Methods

Study 1 was conducted as an initial study where we wanted to find out which conditions were interesting in particular. Accordingly, we targeted to reveal even small effects. We further stressed the detection of effects against testing null-effects (focusing on α and not β). For the initial study, we had no knowledge of how strong our target variables (e.g., attractiveness and sympathy) correlated. Accordingly, we set all the pre-defined correlations to relatively weak intercorrelations. With an α-level of 0.05, a power of 0.80 and an effect size to be able to detect *f* = 0.10 we obtained a minimum total sample size of 161.

#### Participants

One Hundred and seventy two observers participated in the online based study (134 female; *M* = 25.2 years, *SD* = 8.3, range 18–61 years) on voluntary basis. Data were collected using the online survey tool “SoSci Survey” (Leiner, 2014). Most of the recruited participants were students of the University of Bamberg and gained course credit to fulfill course requirements. All other participants were recruited by online announcements (e.g., Facebook groups). All participants were naïve to the aim of the study and were not familiar with the presented faces.

#### Materials

In order to ascertain the precise orientation of a face with respect to the vantage point of the camera, we selected 3D face scans (Di3D-technology) of 14 human models (7 female, aged *M* = 25.0 years, *SD* = 3.3, range 20–31 years). We aligned these models with respect to a virtual camera and created 2D images of the faces corresponding to a camera position aligned with the inter-ocular point and perpendicular to the vertical axis of the face. We then rendered the image from seven camera perspectives (see Table 2) using Autodesk 3ds™ Max 2017 (note: the perspectives were all defined in terms of the face owner's view): *above_30°_*(“from above,” which is equivalent to a camera raised and tilted by 30°), *below_30°_*(“from below,” which is equivalent to a camera lowered and tilted by 30°), *15° left* (rotated, which is equivalent to a camera located 15° to the left side of the face: we refer to this manipulation as *left hemiface_15°_*), *30*° *left* (rotated, which is equivalent to a camera located 30° to the left side of the face: we refer to this manipulation as *left hemiface30*°), *15*° *right* (rotated, which is equivalent to a camera located 15° to the right side of the face: we refer to this manipulation as *right hemiface15*°), *30*° *right* (rotated, which is equivalent to a camera located 30° to the right side of the face: we refer to this manipulation as *right hemiface30*°), and *0*° (frontal view, which is equivalent to a frontal snapshot). The use of these seven perspectives was inspired by a study of Schneider et al. (2012) who only used gradations of 30° which we extended by using more finely graduated levels of 15° levels (0°, 15°, 30°). We refer to this manipulation as *viewing perspective* in the following. Please see example stimulus with the respective manipulation in Table 2.

**Table 2:** Study 1: Mean facial judgments and effect sizes (Cohen's *d*) across different *viewing perspectives* with a focus on differences between the *frontal* condition and the other *viewing perspectives* split by *model gender* (negative values of effect sizes indicate lower judgments compared to the *frontal* condition).

**Dependent variable**	**Gender**	**Below_30°_**	**Left hemiface_30°_**	**Left hemiface_15°_**	**Frontal**	**Right hemiface_15°_**	**Right hemiface_30°_**	**Above_30°_**
		** 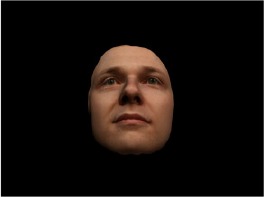 **	** 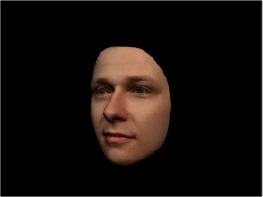 **	** 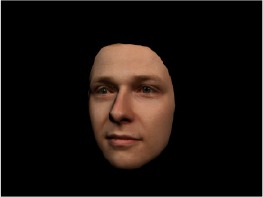 **	** 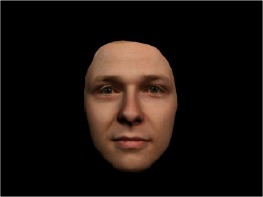 **	** 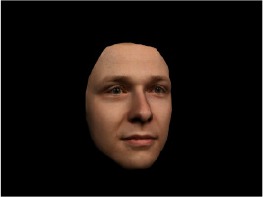 **	** 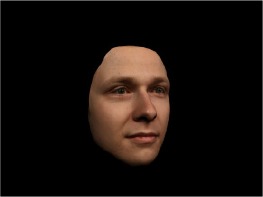 **	** 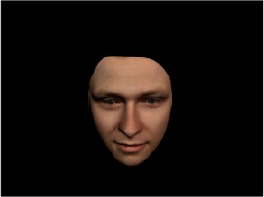 **
Attractiveness	♀	2.95	3.54^**^ (1.33)	3.65^***^ (1.58)^**^	2.84	3.21^*^ (0.58)	3.54^**^ (1.12)	2.68
	♂	3.04	3.59^*^ (0.74)	3.70^**^ (0.95)	3.06	3.38^*^ (0.43)	3.52^*^ (0.55)	2.81
	Total	3.00	3.57^**^ (1.01)	3.68^***^ (1.25)	2.95	3.29^*^ (0.52)	3.53^**^ (0.81)	2.75
Helpfulness	♀	4.04	4.09	3.97	4.01	4.15	4.21^*^ (0.31)	3.08^***^ (−1.47)
	♂	3.99	3.92	4.00	4.06	4.10	4.21	3.06^***^ (−1.96)
	Total	4.02	4.00	3.99	4.03	4.13	4.21^*^ (0.31)	3.07^***^ (−1.75)
Sympathy	♀	3.85	4.01	4.07	3.84	3.94	4.11	3.49
	♂	3.97	3.98	4.06	3.85	4.10	4.19^*^ (0.49)	3.56
	Total	3.91	4.00	4.07	3.84	4.02	4.15^*^ (0.34)	3.53
Dominance	♀	4.06	3.68	3.68	3.97	3.77	3.97	4.30
	♂	4.14	3.81	3.69	3.81	3.97	3.93	4.04
	Total	4.10	3.75	3.69	3.89	3.87	3.95	4.17
Distinctiveness	♀	3.47	3.73	3.99	3.95	4.03	3.81	4.17
	♂	3.82	3.92	4.20	4.03	4.05	4.02	4.11
	Total	3.64	3.82	4.09	3.99	4.04	3.91	4.14
Intelligence	♀	4.22	4.13	4.27	4.06	4.09	4.46^*^ (0.66)	4.09
	♂	4.20	4.18	4.13	3.99	4.22	4.27	3.85
	Total	4.21	4.16	4.20	4.03	4.16	4.37^*^ (0.59)	3.97
Body weight	♀	75.90^**^ (1.13)	70.36	69.93	71.91	70.73	70.81	65.26^**^ (−1.89)
	♂	80.00^***^ (1.75)	71.95	72.54	73.62	71.99	71.11^**^ (−0.75)	68.23^**^ (−1.72)
	Total	77.95^***^ (1.36)	71.16^*^ (−0.48)	71.24	72.76	71.36^*^ (−0.46)	70.96^**^ (−0.56)	66.75^***^ (−1.75)

Significances were marked as bold values (

* *p ≤ 0.05*,

** *p ≤ 0.01*,

*** *p ≤ 0.001)*.

*Additionally, direct comparisons between left and right hemifaces were calculated and significant effects were marked with gray boxes corresponding to their effect sizes [see Cohen (1988): medium gray indicates medium large effects (Cohen's d = [0.5–0.8]) and dark gray indicates large effects size (Cohen's d > 0.8)]. Light gray column in the middle highlights the frontal condition. Example stimuli in the first row: Please note that we obtained consent to publish the individual's face in the present study*.

#### Procedure

The study had two factors: *model gender* (gender of the shown face) and *viewing perspective*, with the dependent variable (rating of personality variables: *attractiveness, helpfulness, sympathy, dominance, distinctiveness, intelligence*, and the associated *body weight*) as the subordinate orders. Factor levels were blocked and their sequences were counterbalanced across participants. This resulted in 2 [gender of model] × 7 [viewing perspective] × 7 [personality dimensions] = 98 trials. Each picture was presented in color on a black background and was standardized to a size of 600 × 450 pixels. Due to the fact that the study ran online, the actual size on the display could not be fully controlled. However, we asked the participants to avoid the use of a mobile device (such as mobile phones and tablets). Furthermore, we kindly asked the participants to use the full screen mode of their browser to reduce destructing visual cues.

For each stimulus, participants provided a rating (on a 7-point Likert scale) or body weight judgment (in kilograms) based on their individual, subjective and spontaneous impression, respectively (by presenting an initial sentence like e.g., “*I perceive the shown face as…*”). The scale ranged from “less” to “very” (e.g., “attractive”). For the variable *distinctiveness*, we additionally referred to the aforementioned definition: “*a distinct face/person is remarkable standing out from a crowd of other faces/persons*.” With respect to the perceived body weight, the initial sentence was “*Please judge the perceived body weight of the shown person in kilograms (in whole numbers)*.” Each trial started with a fixation cross followed by a blank screen and the target face until a response on the keyboard was made. The whole procedure lasted ~15 min.

### Results

One of the main goals of this study was to understand the nature of selfies in contrast to conventional frontal portraits, such as current passport photos in the European Union. Accordingly, analyses focused on potential differences between the *frontal* condition and the other *viewing perspectives*. Data were analyzed with a two-factorial repeated-measures analysis of variance (rmANOVA) with the within-subject factor *viewing perspective* and the between-subject factor *model gender*. An univariate approach with Huynh-Feldt correction (Huynh and Feldt, 1976) for the degrees of freedom (*df*) was used (correction factor ε), which should be applied if ε is >0.75 (Girden, 1992). Furthermore, it shows good control of the Type I error rate (Oberfeld and Franke, 2013). The original value of the *df* is reported. Partial η^2^ ($\eta_{p}^{2}$) is reported as a measure of association strength. An α-level of 0.05 was used for all analyses reported in this paper and all reported *p*-values are two-tailed. Pairwise comparisons and respective Cohen's *d* were additionally calculated (see Table 2). Further analyses were conducted with a focus on the simple main effects. All assumptions of a repeated measurement ANOVA were sufficiently fulfilled: independence of observations, normality of distribution of residuals as well as the homoscedasticity across and within all groups. All analyses were conducted by using RStudio (ver. 0.99.903) for Mac.

Regarding the *attractiveness* ratings, we found a significant main effect of *viewing perspective, F*_(6, 72)_ = 19.80, *p* < 0.0001, η_*p*_^2^ = 0.62, ε = 0.91. In comparison to *frontal* snapshots (*M*_*frontal*_ = 2.95, *SD*_*frontal*_ = 0.73), further analyses revealed that sided snapshots were rated as significantly more attractive. However, this effect was more pronounced for snapshots of the *left hemiface* compared to the *right hemiface*, but only for the *15*° *left* condition (direct comparison: *M_15°left_* = 3.68, *SD_15°left_* = 0.38 vs. *M_15°right_* = 3.29, *SD_15°right_* = 0.60; *d* = 0.77). Although the *left hemiface* affected both genders, the effect was more pronounced for *female* faces (direct comparison: *M_15°leftfemalefaces_* = 3.65, *SD_15°leftfemalefaces_* = 0.35 vs. *M_15°rightfemalefaces_* = 3.21, *SD_15°rightfemalefaces_* = 0.62; *d* = 0.89 and *M_15°leftmalefaces_* = 3.70, *SD_15°leftmalefaces_* = 0.43 vs. *M_15°rightmalefaces_* = 3.38, *SD_15°rightmalefaces_* = 0.62; *d* = 0.60). So, on average, showing the left cheek seems to be slightly more appealing, see Table 2.

Analyses for *helpfulness* revealed a significant main effect of *viewing perspective, F*_(6, 72)_ = 29.53, *p* < 0.0001, η_*p*_^2^ = 0.711, ε = 1.00. Interestingly, in comparison to *frontal* snapshots (*M*_*frontal*_ = 4.03, *SD*_*frontal*_ = 0.64), pairwise comparison revealed that faces photographed from a higher viewing perspective (*above*30°) were rated as significantly *less* helpful, (*M_above30°_* = 3.07, *SD_above30°_* = 0.44, *d* = −1.75) across female and male faces, suggesting a body height dependent effect on the perception of *helpfulness* (see Table 2). For snapshots of the *right hemiface30*° we additionally found a small effect for only female faces (*M_30°right_* = 4.21, *SD_30°right_* = 0.60, *d* = 0.31), see Table 2. However, direct comparisons of *left* vs. *right hemifaces* revealed no effect. Further analyses for the variable *sympathy* revealed a main effect of *viewing perspective, F*_(6, 72)_ = 5.70, *p* < 0.0001, η_*p*_^2^ = 0.322, ε = 0.87. More specifically, we found a small effect for snapshots of the *right hemiface30*° (*M_30°right_* = 4.15, *SD_30°right_* = 0.77, *d* = 0.40), see Table 2. This effect was slightly more pronounced in male faces (*M_30°right_* = 4.19, *SD_30°right_* = 0.63, *d* = 0.49). Again, direct comparisons of *left* vs. *right hemifaces* revealed no effect. Analyses for the variable *intelligence* revealed a small but significant main effect of *viewing perspective, F*_(6, 72)_ = 2.39, *p* = 0.041, η_*p*_^2^ = 0.166, ε = 0.94. In comparison to *frontal* snapshots (*M*_*frontal*_ = 4.03, *SD*_*frontal*_ = 0.72), analyses revealed that the *right hemiface30*° (*M_30°right_* = 4.37, *SD_30°right_* = 0.40, *d* = 0.59) was rated as slightly *more* intelligent. Direct comparisons of *left* vs. *right hemifaces* revealed no effect.

Regarding the body weight judgments, we found a strong effect of *viewing perspective, F*_(6, 72)_ = 31.10, *p* < 0.0001, η_*p*_^2^ = 0.722, ε = 0.95, replicating the results reported by Schneider et al. (2012). In comparison to the *frontal* condition (*M*_*frontal*_ = 71.91, *SD*_*frontal*_ = 3.41), the associated body weight for faces photographed from a lower perspective (*M_below30°_* = 77.95, *SD_below30°_* = 4.10, *d* = 1.36) was rated as significantly higher than for faces photographed from a higher perspective (*M_above30°_* = 66.75, *SD_above30°_* = 3.37, *d* = −1.75), see Table 2. We further found that snapshots of the *right hemiface* produced slightly lower body weight judgments (*M_15°right_* = 71.36, *SD_15°right_* = 2.60, *d* = −0.46 and *M_30°right_* = 70.96, *SD_30°right_* = 2.89, *d* = −0.56). Furthermore, left cheek views (showing the *left hemiface*) also produced significantly lower associated body weight assessments (*M_30°left_* = 71.16, *SD_30°left_* = 3.11, *d* = −0.48). Other *viewing perspectives* had no effect on the associated body weight. We did not find any effects for the variables *dominance* or *distinctiveness*.

### Discussion

The main goal of Study 1 was to investigate the potential effects of different *perspectives* on the perception of a given face, compared to classical frontal portrait photos. In Study 1, we let our participants rate person-related variables across different viewing perspectives on the basis of faces. We were able to show that in the case of *attractiveness* ratings, the perspective of the camera had a significant effect. This effect was especially positive for presentations of the *left hemiface* and more distinct for female faces (in contrast to male faces) what is in line with findings by Sitton et al. (2006), although others, e.g., Dunstan and Lindell (2012) did only found this effect for male faces. This optimization possibility is seemingly often used in the field when people take a selfie: Here, people tend to show a side bias (mostly showing the left cheek)—interestingly, the *left hemiface* has a significant effect on the perception of (positive) emotion (see McManus and Humphrey, 1973; Bruno and Bertamini, 2013; Lindell, 2013a,b; Low and Lindell, 2016). However, our results are also in some contrast to our initial hypothesis and also to other research: e.g., Burt and Perrett (1997) used chimeric faces and revealed that the *right hemiface* impacts attractiveness more than the left side. Other research e.g., Zaidel et al. (1995) as well as Dunstan and Lindell (2012) also revealed this right side effect but only for female faces. Furthermore, in our dataset the *right hemiface* also positively affected the perception of attractiveness although this effect was less pronounced. However, in these studies only frontal (partly chimeric) faces were used and stimuli were not rendered from 3D models. Dunstan and Lindell (2012), in contrast, used photographs of human models but with a visible torso and direct gaze toward the camera. In the present study, we decided to use fully rotated faces which were based on photogrammetry which allows the extracting of variable perspectives from one single face model, so that all instances show the very same face at one fixed moment in time. Similarly, Burke et al. (2007) and Schneider et al. (2012) suggested that depth information (which was highly available in our stimuli set) in particular contributes to differences in the perception of a face. In contrast to our hypothesis, we did not find any effects of elevating or lowering the camera, neither for male (lowering the camera) nor for female faces (elevating the camera). However, this is in line with recent research by Baranowski and Hecht (in press) who did not find such an effect in faces of (unknown) actors.

Regarding the variable *helpfulness*, we found a small but significant effect for right sided faces and a clear negative effect for faces shown from a higher vantage point, suggesting a height-dependent effect of viewing perspective on perceived helpfulness. Regarding the *above30*° condition, which is equivalent to a taller person looking down on a smaller person, recent research revealed that taller persons are associated with greater leadership skills (Re et al., 2012, 2013). From this point of view, you may expect that smaller persons indeed rely on the helpfulness of the respective leader instead of being more helpful themselves. Accordingly, persons seen from above, such as typically smaller persons, might be assessed as less helpful—or even more precisely, as being potentially less helpful. Interestingly, we found such a perspective-relevant effect on helpfulness only with faces that are observed from above, but we failed to document an effect of higher helpfulness with faces observed from below. Furthermore, showing the *right cheek* (compared to the *frontal* condition) positively affects the perception of *helpfulness* especially for female faces. Beside the fact that this effect was rather small and *not* significantly larger for the *left cheek* condition, we could only speculate: similarly, we also found a significant and positive effect for the *right hemiface* on the perceived *intelligence*. Following the results of a recent study by Furnham and Cheng (2015), *intelligence* could be taken as a predictor for *helpful behavior* (as a facet of agreeableness). Accordingly, this may explain the similar pattern of *helpfulness* and *intelligence*. However, the effect of gender as well as the effect of rotation could not be sufficiently explained. A possible explanation for the right-side bias in the perception of *intelligence* is provided by findings that the *right hemiface* is associated with scientific, rational, academic and unemotional concepts (e.g., Nicholls et al., 1999; ten Cate, 2002; Lindell and Savill, 2010; Churches et al., 2012): e.g., in a study ten Cate (2002) presented pictures of professors of the eighteenth century and let participants rate how “scientific” they perceived the respective professor. Accordingly, participants rated the right cheek pictures as more scientific. This finding was further extended by Churches et al. (2012) who found that people intuitively show either the left or the right cheek, depending on what they want to express (scientists of core-sciences such was mathematics, engineering as well as chemistry show their right cheek, whereas scientists of human sciences such as psychology tended to show the left cheek).

With respect to the perceived *sympathy*, we found a significant and positive effect for right sided snapshots (showing the right cheek) especially for *male* faces (compared to the *frontal* condition). However, direct comparison of *left* vs. *right* hemifaces revealed no significant difference. Accordingly, our results might contrast past findings according to which the *left* hemiface is perceived as more emotional (see e.g., Sackeim et al., 1978; Zaidel et al., 1995; Nicholls et al., 2002; Jones et al., 2012; Lindell, 2013a,b; Low and Lindell, 2016). However, to the author's knowledge, there is no investigation on the perception of *sympathy* with respect to viewing perspective. Moreover, we assume that *sympathy* is only a single facet of the entire and complex construct of emotion. Thus, the pattern of our data leads to the speculation that it does *not* contradict past findings, since the perception of emotions is not homogenously unilaterally affected.

With respect to the perception of the associated *dominance*, past research revealed that raising the head improves the perception of it (e.g., Otta et al., 1994; Mignault and Chaudhuri, 2003; Chiao et al., 2008; Rule et al., 2012). Similarly, Burke and Sulikowski (2010) demonstrated a clear association between upward postures and perceived masculinity. Thus, we expected higher ratings for upward-pitched faces and lower ratings for downward-pitched faces, compared to the *frontal* condition. However, we did not find this effect in our sample. Moreover, there was not even any significant difference between upward vs. downward pitched faces. Calling our results into question, we suggest that *cervical* cues (e.g., the visibility of a neck) are essential for the perception of dominance (keep in mind that in the aforementioned studies, the neck was visible). Additionally, the human *trapezius muscle* (a large muscle that extends longitudinally from the occipital bone to the lower thoracic vertebrae and laterally to the spine of the shoulder blade) is more visible and especially the *longus colli muscle* (the long muscle of the neck) is in more tension in the case of raised heads. Most notably with male bodies, Frederick and Haselton (2007) demonstrated that perceived *dominance* is strongly dependent on the perceived muscle mass. Our set of stimuli was limited to neckless faces only. Accordingly, important cues to muscle mass and *dominance* were not accessible.

Considering past research, the effects that were mainly investigated were of *viewpoint* on recognition processes, relating to *distinctiveness*. It is suggested that distinctive faces are recognized better than ones that are more typical in their appearance (in the sense of Valentine, 1991 so-called *Multidimensional Face Space Model*): Typical faces are densely located near the centroid of this face space, hence there is a high potential for confusion; whereas distinctive faces are less densely clustered (e.g., Valentine, 1991; Newell et al., 1999). Regarding to our study, research revealed that in cases of *unfamiliar* face processing, changes due to (planar) rotation (i.e., a rotation called “roll”) makes face recognition harder. In fact such a kind of rotation disrupts featural (e.g., Carbon and Leder, 2006; Stephan and Caine, 2007; Akselrod-Ballin and Ullman, 2008) as well as “configural processing” (e.g., Carbon and Leder, 2006; Favelle et al., 2011) and “holistic processing” (Tanaka and Farah, 1993; Leder and Carbon, 2005; Goffaux et al., 2009; but see Richler et al., 2011). In the present study we addressed the much-less-investigated case of faces rotated in terms of “yaw” and “pitch.” Furthermore, relating to face recognition, research revealed an interaction between *distinctiveness* and *viewing perspective*. More specifically, it is suggested that the visibility of distinctive parts of a face varies across different *viewing perspectives*, hence recognition performance is dependent on the availability of these parts: distinctive facial features could be invisible in faces which are presented in profile (e.g., Valentin et al., 1999, 2001). However, direct potential effects of *viewing perspective* on *distinctiveness* have not yet been investigated. In our study, we could not find any effects of *perspective* on *distinctiveness*; probably the extent of utilized deviations from the *frontal* perspective was just not large enough to find any effects. This would be in accordance with previous research wherein robust face processing of configural aspects was documented up to a (planar) rotation of about 60° from the frontal-upright orientation (Carbon et al., 2007).

In Study 1, we were able to replicate the so called “*height-weight illusion*” (first mentioned by Schneider et al., 2012) whereby faces seen from a higher viewing perspective are associated with a significantly lower body weight compared to faces seen from a lower viewing perspective. This advantage was slightly more pronounced in faces showing their right cheek (*right hemiface*). This finding is in accordance with research that revealed a preference for sided faces (e.g., Bruno and Bertamini, 2013; Yeh and Lin, 2014). Furthermore, it underlines the correlation between the perception of facial mass (and respective body weight), and perceived attractiveness (e.g., Tovee et al., 1998, 1999, 2006; Swami et al., 2006, 2010; Coetzee et al., 2009, 2010).

## Study 2

Study 1 revealed that *perspective* has an impact on facial judgments, especially for body weight judgments (previews findings are reported by e.g., Schneider et al., 2012, 2013); other postulated effects were less pronounced or absent. However, the used viewing perspectives of Study 1 are sometimes found with selfies but some additional ones are even more typical of the selfie style (see e.g., Bruno et al., 2014). Just imagine that you are going to take a selfie on your next trip. It is unlikely that you will only rotate your mobile phone rigidly around one axis, but typically you will use a combination of such rotations. Accordingly, the aim of Study 2 was to examine the impact of typical *perspectives* of selfies on facial judgments.

For study 2, we focused on medium size effects as the study was framed in a more applied context expecting rather more noise and less signal. Accordingly, we adjusted our pre-sets in terms of effect size (*f* = 0.25) and power (1 − β = 0.95), yielding a needed total sample size of 45.

### Method

#### Participants

Sixty-seven observers participated in the online-based study (52 female; *M* = 24.3 years, *SD* = 3.6, range 19–38 years) on a voluntary basis. Data were collected using the online study tool “SoSci Survey” (Leiner, 2014). Method of recruiting participants was the same as in Study 1. All participants were naïve to the aim of the study; none of them participated in Study 1; they were not familiar to the presented faces.

#### Materials

The stimulus material of Study 2 was the same as in Study 1, with the difference that we changed the used *viewing perspectives* toward an even more selfie-esque style by combining tilted and rotated camera conditions (see Carbon, 2017). As a result, we got the following seven *viewing perspectives* (see Table 3):

- *above_30°_*, *below_30°_*(both, *above_30°_* and *below_30°_*as in Study 1),
- *above_30°left_*(combination: elevated/rotated, which is equivalent to a raised and tilted camera *plus* a camera located *30*° to the left side of the face) *below_30°left_* (combination: lowered/rotated, which is equivalent to a lowered and tilted camera *plus* a camera located *30*° to the left side of the face), *above_30°right_*(combination: elevated/rotated, which is equivalent to a raised and tilted camera *plus* a camera located *30*° to the right side of the face), *below_30°right_*(combination: lowered/rotated, which is equivalent to a lowered and tilted camera *plus* a camera located *30*° to the right side of the face), and *0*° (frontal view, which is equivalent to a frontal snapshot).

**Table 3:** Study 2: Mean facial judgments and effect sizes (Cohen's *d*) across different *viewing perspectives* with a focus on differences between the *frontal* condition and the other *viewing perspectives* split by *model gender* (negative values of effect sizes indicate lower judgments compared to the *frontal* condition).

**Dependent variable**	**Gender**	**Below_30°_**	**Below_30°left_**	**Above_30°left_**	**Frontal**	**Above_30°right_**	**Below_30°right_**	**Above_30°_**
		** 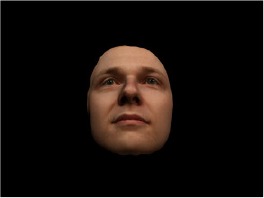 **	** 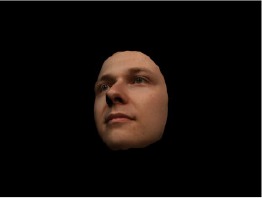 **	** 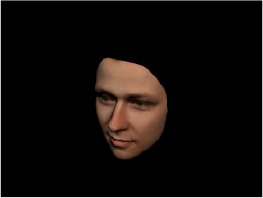 **	** 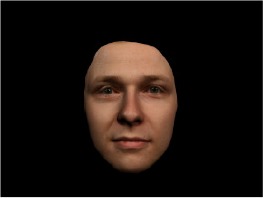 **	** 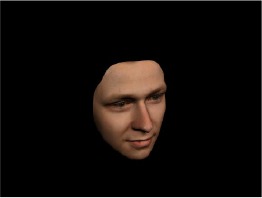 **	** 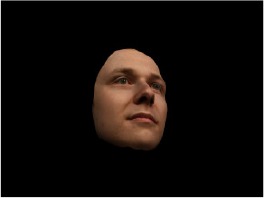 **	** 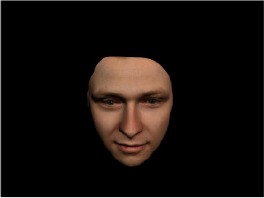 **
Attractiveness	♀	3.90	3.30	4.29	3.65	4.12	2.25^**^ (−1.44)	3.32
	♂	2.93	2.17	4.16^**^ (1.77)	2.79	4.10^*^ (1.67)	2.25	2.64
	Total	3.42	2.73	4.23^**^ (1.20)	3.22	4.11^*^ (1.02)	2.25^**^ (−1.11)	2.98
Helpfulness	♀	4.02	4.07	4.15	4.30	3.98	3.64	2.66^***^ (−2.26)
	♂	3.70	2.96	3.91^*^ (1.23)	3.04	4.41^***^ (1.62)	3.30	2.39
	Total	3.89	3.52	4.03	3.67	4.20^*^ (0.53)	3.47	2.52^**^ (−1.41)
Sympathy	♀	4.05	4.01	4.15	3.96	4.35	3.49	4.48
	♂	3.56	3.11	3.65	3.06	4.31	3.50	3.61
	Total	3.81	3.56	3.90	3.51	4.33	3.49	4.04
Dominance	♀	4.42	3.66	3.40	3.75	3.68	3.71	3.07
	♂	4.30	4.18	4.05	4.03	4.53	4.72	4.00
	Total	4.36	3.92	3.73	3.89	4.10	4.21	3.54
Distinctiveness	♀	4.76	4.09	3.84	3.92	4.30	4.92	4.44
	♂	4.38	3.90	3.94	4.08	4.10	4.55	3.53
	Total	4.57	3.99	3.91	4.00	4.20	4.74	3.98
Intelligence	♀	4.55	4.68	4.07	4.27	4.39	4.23	4.32
	♂	3.63	3.84	3.67	4.02	4.54	4.14	3.83
	Total	4.09	4.26	3.87	4.14	4.47	4.19	4.08
Body weight	♀	66.94^**^ (3.20)	63.28^**^ (2.86)	50.99^**^ (−3.65)	57.96	51.14^***^ (−3.51)	61.72^**^ (2.19)	51.77^**^ (−2.68)
	♂	83.48^***^ (2.00)	79.58^***^ (2.12)	65.61^***^ (−2.11)	73.55	63.12^***^ (−3.47)	79.76^***^ (2.10)	64.94^***^ (−2.82)
	Total	75.21^***^ (1.03)	71.43^***^ (0.66)	58.30^***^ (−0.89)	65.75	57.13^***^ (−1.13)	70.74^***^ (0.55)	58.35^***^ (−0.93)

Significances were marked as bold values (

* *p ≤ 0.05*,

** *p ≤ 0.01*,

*** *p ≤ 0.001)*.

*Additionally, direct comparisons between the left and right hemiface plus above and below conditions were calculated, but we did not find any significant effect. Example stimuli in the first row: Please note that we obtained consent to publish the individual's face in the present study. Light gray column in the middle highlights the frontal condition*.

#### Procedure

The procedure was the same as in Study 1.

### Results

In Study 2 we focused again on the impact of different perspectives on several person-related variables, always with the *frontal* perspective as the base condition. To be able to optimally compare the results between both studies, we followed the same strategy of analyses (see details above).

Regarding the *attractiveness* ratings, we found a significant main effect of *viewing perspective, F*_(6, 72)_ = 11.75, *p* < 0.0001, η_*p*_^2^ = 0.495, ε = 1.00. In comparison to *frontal* snapshots (*M*_*frontal*_ = 3.22, *SD*_*frontal*_ = 0.90), analyses revealed that elevating and rotating the camera had a large positive effect on *attractiveness* (*M_above30°left_* = 4.23, *SD_above30°left_* = 0.78, *d* = 1.20 and *M_above30°right_* = 4.11, *SD_above30°right_* = 0.83, *d* = 1.02). In both cases (snapshots of the *left* and *right hemiface*), the effect was more pronounced for male faces (*M_above30°left_* = 4.16, *SD_above30°left_* = 0.96, *d* = 1.77 and *M_above30°right_* = 4.10, *SD_above30°right_* = 0.97, *d* = 1.67). In contrast, lowering and rotating the camera had a negative effect on *attractiveness* (*M_below30°right_* = 2.25, *SD_below30°right_* = 0.86, *d* = −1.11). This effect was more pronounced for female faces (*M_below30°right_* = 2.25, *SD_below30°right_* = 0.95, *d* = −1.44), see Table 3.

Analyses for the variable *helpfulness* revealed a significant main effect of *viewing perspective, F*_(6, 72)_ = 7.95, *p* < .0001, η_*p*_^2^ = 0.398, ε = 1.00. Similarly to Study 1, in comparison to *frontal* snapshots (*M*_*frontal*_ = 3.67, *SD*_*frontal*_ = 1.08), faces photographed from a higher viewing perspective (*above30*°) were rated as significantly *less* helpful (*M_above30°_* = 2.52, *SD_above30°_* = 0.40, *d* = −1.41). This effect was particularly large for *female* faces (*M_above30°_* = 2.66, *SD_−30°_* = 0.45, *d* = −2.26), see Table 3. Elevating the camera, however, did *not* have an effect. Specifically, for male faces, a *combination* of elevation and rotation of the camera (*above_30°left_* and *above_30°right_*) led to significantly higher *helpfulness* ratings (*M_above30°left_* = 3.91, *SD_above30°left_* = 0.55, *d* = 1.23 and *M_above30°right_* = 4.41, *SD_above30°left_* = 0.85, *d* = 1.62), suggesting an interaction of gender and viewing perspective. Regarding the variable *sympathy*, we found higher ratings for male faces which were photographed from a higher viewing perspective and rotated by 30°_right_. However, this effect was not significant; see Table 3.

Regarding *body weight* judgments, we replicated the height-weight illusion (Schneider et al., 2012) which was also found in Study 1, see Table 3. Furthermore, compared to the *frontal* condition (*M*_*frontal*_ = 65.75, *SD*_*frontal*_ = 8.65), lowering *plus* rotating the camera produced significantly higher body weight judgments (*M_below30°left_* = 71.43, *SD_below30°left_* = 8.53, *d* = 0.66 and *M_below30°right_* = 70.74, *SD_below30°right_* = 9.44, *d* = 0.55). Interestingly these conditions (*below_30°left_* and *below_30°right_*), were still slightly lower than the pure *above30*°condition without a horizontal rotation (*M_below30°_* = 75.21, *SD_below30°_* = 9.71, *d_below30°left_* = −0.41 and *d_below30°left_* = −0.47). We could also detect that elevating and rotating the camera indeed produced significantly lower body weight judgments (*M_above30°left_* = 58.30, *SD_above30°left_* = 8.04, *d* = −0.89 and *M_above30°right_* = 57.13, *SD_above30°right_* = 6.41, *d* = −1.13). Nevertheless, additional horizontal rotation of the camera did not significantly enhance the effect of height-weight illusion. In line with Study 1, we did not find any effects for the variables *sympathy, dominance, distinctiveness*, or *intelligence*.

### Discussion

The aim of Study 2 was to examine whether more selfie-specific *viewing perspectives* have an even more pronounced effect on facial judgments. Accordingly, in Study 2, we let participants rate personality variables across different viewing perspectives on the basis of faces. In accordance with the findings of Study 1, we could show that in case of *attractiveness* judgments were positively affected by horizontally rotating and elevating the camera. Similarly to Study 1, this effect was slightly (but not significantly) more pronounced for the left side of the face compared to the right side. We also reported larger effects for male faces compared to female faces. This suggests a clear preference for lateral and elevated snapshots. This conclusion is supported by findings that elevating the camera *plus* rotating the camera is generally preferred for taking selfies (Yeh and Lin, 2014; Kalayeh et al., 2015). An elevation within pure frontal depictions had *no* effect on attractiveness ratings at all what is in line with Study 1 and findings by Baranowski and Hecht (in press). However, there was a slight (but non-significant) decrease in perceived attractiveness. In the case of the *below*_*right*_ condition (which is equivalent to a view from the right bottom) we found a *decrease* in perceived attractiveness, and this effect was even more pronounced for female faces. Burt and Perrett (1997) as well as Zaidel et al. (1995) argued that the right side of the owner's face positively affects the perception of facial attractiveness. However, this effect had not yet been investigated in combination with a classical selfie-style camera upward tilt. Similarly, it could be shown that facial cues can be taken as a valid predictor of body weight and this highly correlates with the perceived health and attractiveness (Coetzee et al., 2009, 2010).

Regarding the assessment of *helpfulness* in Study 2, we showed that elevating and rotating the camera had a significant and positive effect. Similarly to Study 1, this effect was again slightly more pronounced in faces showing their right cheek (*above_30°right_*). In contrast, we replicated the negative effect of Study 1 (a *frontally* elevated camera: the *above*30° condition is equivalent to a taller person looking downwards on a smaller person). At first sight this contradicts the finding of Study 1, where we argued the typical view of a taller person caused people to assess the viewed person as more helpful. The additional horizontal rotation eliminated this effect. We can only speculate at this point, but in the specific combination of tilting and rotating a camera might have induced a higher rating for helpfulness in Study 2 as this perspective reveals many details of the face and also looks quite realistic—the participants probably perceived a face from this perspective as much more of a real face than would have been the case with a flat picture of a face. The variable *helpfulness* might benefit from such a more holistic capture of a face to a greater extent than other variables.

Regarding the *body weight* judgments, we replicated the height-weight illusion that we also documented for Study 1. From this point of view (Schneider et al., 2012), we expected and found generally higher body weight judgments for lower camera positions and generally lower body weight judgments for elevated camera positions. Surprisingly, in cases of lower camera positions (*below*_*30°left*_ and *below_30°right_*), we were able to show that a further camera rotation slightly reduced the effect of higher body weight judgments and this was significant compared to the *below30*° control condition. This suggests a strong positive rotation effect on perceived body weight, which is in accordance with the findings of Study 1. Similarly, we also found a slight but non-significant advantage in the combination of elevating and rotating the camera. Taken together, elevating the camera produces significantly lower body weight judgments across all conditions. An additional rotation does not sufficiently improve this effect. Lowering the camera produces significantly higher body weight judgments across all conditions. However, an additional rotation has a significant effect on perceived body weight (lower body weight judgments).

## General discussion

The main goal of this study was to reveal the impact of perspective on persons depicted via selfies. In two studies, we revealed clear effects of *perspective* on higher cognitive processes (namely the perception of person-related variables on the basis of facial depictions). Research on selfies has revealed that persons who shoot selfies want to express their mood, their personality and even their lifestyle via selfies, so they try to optimize this information by intuitively adapting the camera position (see e.g., Sorokowska et al., 2016). Previous work documented that in cases of classical portraits there were a lot of compositional suggestions and artificial rules which were applied to gain pictures of high appeal, e.g., the “Golden Ratio Rule” or the “Rule of Thirds” or general placement principles of facial features (see e.g., Tyler, 1998a,b; Westphalen, 2016). However, scientific research is quite far from achieving consistent results about the meaningfulness and effects of these rules in general (e.g., Green, 1995; Höge, 1997; McManus and Weatherby, 1997; McManus and Thomas, 2007; Bertamini et al., 2011). In contrast, regarding the social phenomenon of taking *selfies*, one may find only a small number of suggestions, often in a relative unsystematic way, for taking the “best” selfie (scientificly investigated by e.g., Yeh and Lin, 2014; Kalayeh et al., 2015) and some photographic rules like the “high-angle shot” (e.g., Mamer, 2013). However, there is little knowledge about *whether* and *how exactly* these aspects may have an impact on the perception of a given face. Moreover, there are some hints toward a general deviation from known photographic principles in selfies (Bruno et al., 2014) and the impact of a typical selfie-style *perspective* has yet to be investigated.

Accordingly, our results suggest that *perspective* has a significant impact on the perception of the beholder, especially for attractiveness, helpfulness, sympathy, intelligence, and associated body weight: Study 1 investigated the impact of *viewing perspective* in cases of more classical portraits and revealed that showing the *right cheek* (showing the *right hemiface*) positively affects the perception of attractiveness, helpfulness, sympathy, intelligence and body weight. This finding is in accordance with the finding that the *right side* of the owner's face (*right hemiface*) affects the perception of attractiveness, age and gender (Zaidel et al., 1995; Burt and Perrett, 1997; Dunstan and Lindell, 2012) more than the left side (*left hemiface*) but is in some contrast to findings that emotional aspects can be derived better and more accurately from the *left side* of the owner's face (e.g., Zaidel et al., 1995; Kramer and Ward, 2011; Lindell, 2013a,b; Low and Lindell, 2016). However, with respect to the perceived *attractiveness*, we found comparative lager effects for the *left hemiface*, contrasting past research by others (for instance, Zaidel et al., 1995; Burt and Perrett, 1997; Dunstan and Lindell, 2012; but also see Sitton et al., 2006). It is important to mention that past research (but see Kramer and Ward, 2011) did not use 2D stimuli generated from real 3D face models for that kind of research question. Schneider et al. (2012) suggested that differences in perceptual aspects (e.g., perceived body weight on the basis of faces) are strongly dependent on depth information, hence *viewing perspective* affects respective ratings.

In Study 2, we investigated the effect of more selfie-style *viewing perspectives* (typical combination of camera rotation and camera pitch) and only found effects for attractiveness, helpfulness and body weight. Importantly, elevating and rotating had a positive effect on these variables and was slightly more pronounced for the *right side* of the face on average. Lowering the camera only had negative effects on perceived attractiveness and body weight. Regarding the perceived body weight, an additional rotation of the camera reduced the effect of a lowered/raised camera, supporting previous findings relating to the *height-weight* illusion (Schneider et al., 2012). The rest of the personality-related variables remained unaffected from a statistical point of view, although they showed slightly higher ratings for right-sided and elevated snapshots on a purely numerical basis.

How can the complex data pattern be interpreted? First of all: *Perspective* has a significant impact on the perception of higher-cognitive variables (such as person-related variables) on the basis of faces. Secondly: Effects of *perspective* were in contrast to some past findings (for example, higher effects for the right side of the face on average in Study 2 and larger effects for attractiveness for the *left* side of the face in Study 1 and 2) suggesting that selfies constitute an own class of pictorial presentations of a person. This is supported by the findings of Bruno et al. (2014) showing a systematic deviation from known photographic rules in selfies. Furthermore, our results highlight the importance of the visibility of certain features in facial stimuli, *per se* (e.g., regarding the perception of *dominance*, our results underline the visibility of the neck as an important cue to masculinity and dominance). Thirdly: Interestingly, for most of the variables effects were significant for the 30° head turn (left and right hemiface) images, but not the 15° head turn images. We have at least two reasons for this discrepancy in mind: On the one hand, the 15° rotation is just too similar to the frontal condition, at least to detect any differences from the frontal view by means of the given experimental setting with limited sample sizes which were only capable of revealing effect sizes of small to medium effect sizes but not, for example, very small effects. On the other hand, referring to research papers which systematically varied other kinds of rotation, e.g., planar rotations, we also observed a certain range of rotations for which essential variables did not change [e.g., Carbon et al., 2007 did not detect any significant change of the target variable grotesqueness as well as the reaction time (RT) associated with this assessment]. Fourthly: In contrast to the common standpoint that we are able to make meaningful suggestions about “how to take the perfect selfie,” our results indicate that we are a long way from having any clear references.

We would also like to mention some limitations of this study: Past research revealed that direct vs. averted gazes have a direct impact on the perception of a given face (e.g., Kampe et al., 2001; Ewing et al., 2010). More precisely, these studies revealed that an averted gaze has a negative effect on the perception of attractiveness. However, the effect of the combination of averted head *plus* direct gaze vs. frontal face *plus* averted gaze across different viewing perspectives on the perception of higher cognitive variables (like those we used) has not yet been investigated. In this study, we did not investigate such a combination, which would incidentally be very much in accord with some Renaissance portraits like La Gioconda by Leonardo da Vinci (see details on the perspective of the Mona Lisa in Carbon and Hesslinger, 2013). Future research should address such further settings to enrich the existing knowledge base on selfies. Another weakness of the present study is that we neither could control the actual size of the presented face on the monitor nor the actual viewing distance. Moreover, we must expect that display color, contrast and brightness were not at the same level across all participants. This might affect the perception of a face dramatically. However, the fact that we could replicate the height-weight illusion (Schneider et al., 2012) makes it conjecturable that other effects were relative stable. Similarly, other studies (e.g., ten Cate, 2002; Churches et al., 2012) used relatively unstandardized images that could not be controlled along those variables, and though revealed consistent results.

Despite all the back draws you always face with standardized and systematically varied material, such experimental material can test already small effects which might be tested with more ecologically valid material in the field later on. We hope that our study contributes to the understanding on how perspective can change the assessment of higher cognitive variables. This will help to sensitize selfie-ists how powerful the use of perspective might be in conveying their inner states.

## Ethics statement

The study was reviewed and approved by the Ethics Committee of the University of Bamberg, Germany. Its protocol was approved.

## Author contributions

TS was responsible for collecting and analyzing/interpreting data as well as for writing this manuscript. CC was responsible for supervision (initial idea and experimental design improvements), result interpretation and critical manuscript reviewing. Both authors agree to be accountable for the content of the work.

### Conflict of interest statement

The authors declare that the research was conducted in the absence of any commercial or financial relationships that could be construed as a potential conflict of interest.
